# Reperfusion Does Not Improve Impaired Rapid-Onset Cortical Plasticity in Patients with Severe Stenosis of the Internal Carotid Artery

**DOI:** 10.1371/journal.pone.0041004

**Published:** 2012-07-23

**Authors:** Jonathan List, Johannes Albers, Julia Kürten, Arne Schwindt, Eike Wilbers, Agnes Flöel

**Affiliations:** 1 Department of Neurology, Charité - Universitätsmedizin Berlin, Berlin, Germany; 2 Department of Neurology, University Hospital Münster, Münster, Germany; 3 Department of Vascular Surgery, University Hospital Münster, Münster, Germany; 4 Center for Stroke Research Berlin, Charité - Universitätsmedizin Berlin, Berlin, Germany; 5 Cluster of Excellence NeuroCure, Charité - Universitätsmedizin Berlin, Berlin, Germany; Centre Hospitalier Universitaire Vaudois Lausanne - CHUV, UNIL, Switzerland

## Abstract

**Background:**

Severe stenosis of the internal carotid artery (ICA) has been associated with impaired cognition in patients, but its effect on rapid-onset cortical plasticity is not known. Carotid endarterectomy (CEA) in patients with severe ICA stenosis reduces stroke risk, but the impact on cognition or physiology of the respective hemisphere remains controversial.

**Methods/Results:**

16 patients with severe stenosis of the ICA and 16 age and sex matched controls were included. Rapid-onset cortical plasticity was assessed using the paired-associative stimulation (PAS) protocol. PAS models long-term synaptic potentiation in human motor cortex, combining repetitive stimulation of the peripheral ulnar nerve with transcranial magnetic stimulation of the contralateral motor cortex. Cognitive status was assessed with a neuropsychological test battery. In patients, verbal learning and rapid-onset cortical plasticity were significantly reduced as compared to controls. Identical follow-up tests in 9 of the 16 patients six months after CEA revealed no improvement of cognitive parameters or cortical plasticity.

**Conclusions:**

Decreased rapid-onset cortical plasticity in patients with severe stenosis of the ICA was not improved by reperfusion. Thus, other strategies known to increase plasticity should be tested for their potential to improve cortical plasticity and subsequently cognition in these patients.

## Introduction

Stenosis of the internal carotid artery (ICA) is responsible for about 15 to 20% of all ischemic strokes [1]. Carotid endarterectomy (CEA) in patients with severe ICA-stenosis can reduce stroke risk [2]. While stroke clearly increases the odds for developing dementia [3|, severe ICA-stenosis even in the absence of stroke seems to be associated with cognitive impairment [4]. A number of studies have investigated the influence of CEA on cognition, but no definite conclusion could be reached [5]–[7]. Theoretically, CEA may be beneficial by improving cerebral perfusion and thus enhancing neuronal metabolism, but cognition could also deteriorate due to potential periprocedural microinfarctions and hypoperfusion [8].

In the present study, a group of patients with severe ICA-stenosis was compared to a matched group of control subjects in order to assess parameters of cognition and rapid-onset cortical plasticity. In a follow-up, the impact of CEA on cognition and plasticity was reassessed after 6 months in a subgroup of the patient population. Cognition was determined by a neuropsychological test battery, rapid-onset cortical plasticity by paired associative stimulation (PAS) [9], a non-invasive brain stimulation paradigm used as a model of Hebbian associative long-term potentiation (LTP) of synaptic efficacy in humans [10], [11]. PAS leads to a rapid input-specific increase in corticomotor excitability if the interval between the two associative stimuli is timed to generate near-synchronous events in the motor cortex (“rapid onset cortical plasticity”), and is now a widely used protocol to study plasticity in healthy and disease conditions [12], [13]. Even though measured in the motor cortex, it shares distinct physiologic properties with synaptic LTP, which is assumed to be the synaptic basis of memory and learning [11]. Previous studies have shown reduced LTP-like plasticity in patients with impaired cognitive function and learning ability, like Alzheimer’s disease [14]. Down-Syndrome [15], and even schizophrenia [16]. Furthermore, a parallel reduction of LTP-like plasticity and learning ability in a cognitive task has been demonstrated recently [17]. Our own group also found that patients with severe white matter lesions displayed normal cognitive functions if LTP-like plasticity was preserved or even enhanced [12].

We hypothesized that cognitive function as well as rapid-onset cortical plasticity would be decreased in patients with severe ICA stenosis. We further expected a parallel modulation of rapid-onset cortical plasticity and cognitive function after CEA.

## Methods

### Patients with Severe ICA Stenosis and Controls

16 patients (aged 61±7 years (mean + SD), range 53–75 years, 8 women) with high-grade stenosis of the ICA (>80%, ECST-Criteria [18]; 9 left sided stenoses) were recruited from the outpatient clinic of the Department of Vascular Surgery and the Stroke Unit of the Department of Neurology of the University Hospital of Münster between May 2009 and January 2010. 16 control subjects were selected from a database to match the patients with regard to age and sex (62±9 years, range 42–71 years, 8 women). All patients and controls fulfilled the following inclusion criteria: 1. Normal motor function on neurological examination, 2. No intake of medication that influence the central nervous system, 3. No signs of severe cognitive deficits (Mini Mental State Examination [19] (MMSE) ≥26).

Beck’s depression inventory [20] (BDI) was administered to assess depressive symptoms. All patients and controls participated in baseline neuropsychological testing and the PAS protocol. Patients then underwent subsequent CEA within one week. 9 patients received a follow-up investigation 6 to 9 months after CEA (PAS, neuropsychological testing). All participants were right-handed, except one patient according to the Edinburgh Handedness Inventory [21].

The study was approved by the local Institutional Review Board in accordance with the declaration of Helsinki on the use of human subjects in experiments. Each participant gave written informed consent.

### Experimental Procedures

#### Neuropsychological testing

Verbal learning and verbal memory were assessed with the German version of the Rey Auditory Verbal Learning Test (AVLT), working memory with the digit span of the Wechsler Memory Scale (WMS), and verbal fluency with the Regensburg word fluency test (RWT) [22].

#### TMS

Transcranial magnetic stimulation (TMS) was delivered through a figure-of-eight shaped coil (9 cm outer diameter of each wing) connected to a Magstim 200 stimulator (Magstim, Whitland, Dyfed, UK).

Participants were seated in a reclining chair. The coil was held tangential to the scalp with the handle pointing backward at an angle of 45° to the interhemispheric fissure. The optimal position (“hot spot”) of the coil was the cortical representation area of the right abductor digiti minimi (ADM) muscle. On the “hot spot” a moderately suprathreshold stimulation intensity was leading to visible abduction of the little finger. The “hot spot” was then marked with a waterproof pen on the scalp of the subject. Motor evoked potentials (MEP) of the ADM muscle were recorded via surface EMG activity using Ag/AgCl surface electrodes in a belly-tendon-montage. Raw MEP-signals were amplified and digitized and then stored on a laboratory computer for later offline analysis. The bandpass filter was 100 Hz to 10 kHz (Portable Keypoint, Dantec, Bristol, UK). Data was digitized at an analog-to-digital rate of 5 kHz.

At optimal coil position, resting motor threshold (rMT) was defined as the stimulus intensity (in % of maximun stimulator output) which was required to produce a MEP of the ADM muscle of at least 50 µV in at least five of ten consecutive trials.

#### Paired associative stimulation (PAS)

PAS consisted of electrical stimulation of the ulnar nerve (300% of individual sensory threshold) contralateral to the ICA stenosis, combined with TMS over the motor cortex ipsilateral to the ICA stenosis. In controls, TMS was performed over the left motor cortex. We used the “hot spot” of the abductor digiti minimi muscle (ADM) to evoke MEPs with a peak-to-peak amplitude of 0.5 to 1 mV in the relaxed targeted muscle. Peripheral stimulation of the ulnar nerve in combination with TMS to the hot spot of the ADM was chosen instead of stimulation of the median nerve in combination with TMS to the hot spot of the adductor pollicis brevis muscle, because previous studies have shown slightly more pronounced PAS-effects with the first protocol compared to the latter [23]. 90 pulses were delivered repetitively (0.1 Hz) with an inter-stimulus interval of 25 milliseconds, which has been shown to be optimal for inducing increase in motor cortex excitability [9]. MEPs were assessed before PAS (baseline), immediately after PAS (T0), 15 minutes after PAS (T15), and 30 minutes after PAS (T30). For each time point, MEPs were induced 20 times with a frequency of 0.1 Hz. MEP-amplitudes of each time point were then averaged and normalised to the MEP amplitude at baseline for each subject. In order to maintain a standardised level of attention during the PAS intervention, subjects were instructed to stay alert, voluntarily relax the hand, where MEPs were elicited, and count the number of ulnar nerve stimulations.

### Statistical Analysis

Statistical analyses were performed using the free statistical software R [24]. No significant deviations from a normal distribution were found for any of the dependent measures using the Kolmogorov-Smirnov test of normality (set to p<0.05) prior to data analysis.

Demographic and clinical characteristics as well as raw data of the neuropsychological test results of the patient and the control group were compared using two-tailed unpaired t-tests.

Individual PAS-induced plasticity was assessed by taking the grand average of MEPs of each subject measured at time point T0, T15 and T30, normalized to the baseline MEP (MEAN-LTP). Values >1 indicate responders to PAS, whereas values ≤1 indicate non-responders to PAS [25]. Patients and controls were compared using two-tailed unpaired t-tests (primary outcome). To test the time-line of effect of PAS, repeated measures ANOVAs (ANOVA_RM_) were employed with TIME as the repeated measure (baseline, T0, T15, T30) and the between-subject factor GROUP (patients, controls). Post hoc testing was conducted using two-tailed unpaired t-tests.

Effects of CEA on neuropsychological test results as well as TMS-measurements were evaluated by comparing test results before and 6 months after CEA by paired t-tests. TMS data are reported as mean ± standard error of the mean (SEM). P-values for statistical significance were set to p<0.05.

## Results

### Comparison of Patients and Controls

#### Demographical data/baseline characteristics

Demographical and baseline characteristics of patients and controls are listed in **Table 1**. Patients and controls did not differ with regard to age, gender, years of education, or BDI (all ps >0.1). MMSE was significantly lower in patients compared to controls (p = 0.02), but still clearly above 26 points in the patient group.

**Table 1:** Demographical data and baseline characteristics in patients and controls.

	Patients(mean ± SD)	Controls(mean ± SD)	p
**Age**	61±7	62±9	0.72
**Gender**	8 F, 8 M	8 F, 8 M	
**Years of education**	11.6±2.5	13.2±3	0.11
**Handedness (Oldfield)**	87.5±50	95±10	0.56
**MMSE**	28±1	29±1	0.02
**BDI**	7±4	6±5	0.55

MMSE: Mini mental state examination, BDI: Beck’s depression inventory, SD: standard deviation; groups were compared using unpaired t-tests.

#### Cognitive testing at baseline

Differences in neuropsychological test scores are shown in **Table 2**. Verbal learning performance was significantly worse in patients compared to controls (p = 0.03), while working memory, verbal episodic memory (delayed recall of the AVLT), and verbal fluency were comparable between both groups.

**Table 2:** Neuropsychological test scores in patients and controls.

Test	Patients (mean test results ± SD)	Controls (mean test results ± SD)	p
**Verbal learning (AVLT (sum 1–5))**	44.1±11.1	52.5±9.4	0.03
**Verbal memory (AVLT, delayed recall)**	8.9±2.9	10.5±2.9	0.13
**Working memory (WMS digit span, sum forward + backward)**	13.3±3.1	14.3±2.7	0.36
**Verbal fluency**	17.8±9.6	21.5±8.2	0.3

AVLT: Auditory verbal learning test, WMS: Wechsler memory scale; SD: standard deviation; groups were compared using unpaired t-tests.

#### TMS measurements

**Figure 1:**
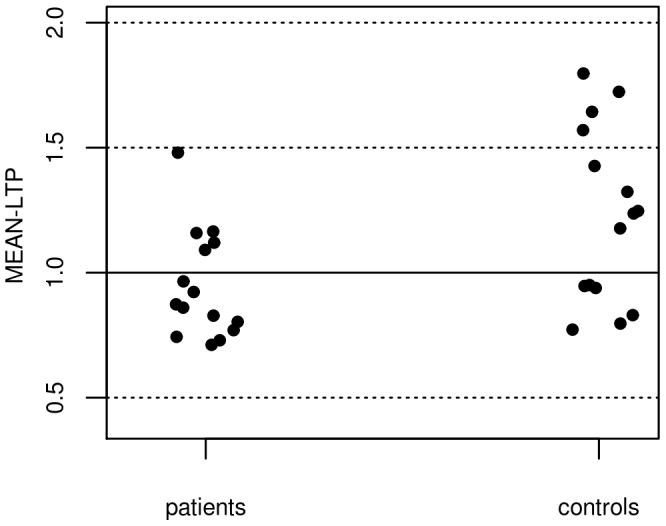
Mean MEP-changes following PAS in patients and controls. MEAN-LTP was assessed by the grand average of MEPs at time point T0 to T30 after PAS, normalized to the baseline MEP. Values >1 indicate responders, whereas values ≤1 indicate non-responders to PAS.

**Figure 2:**
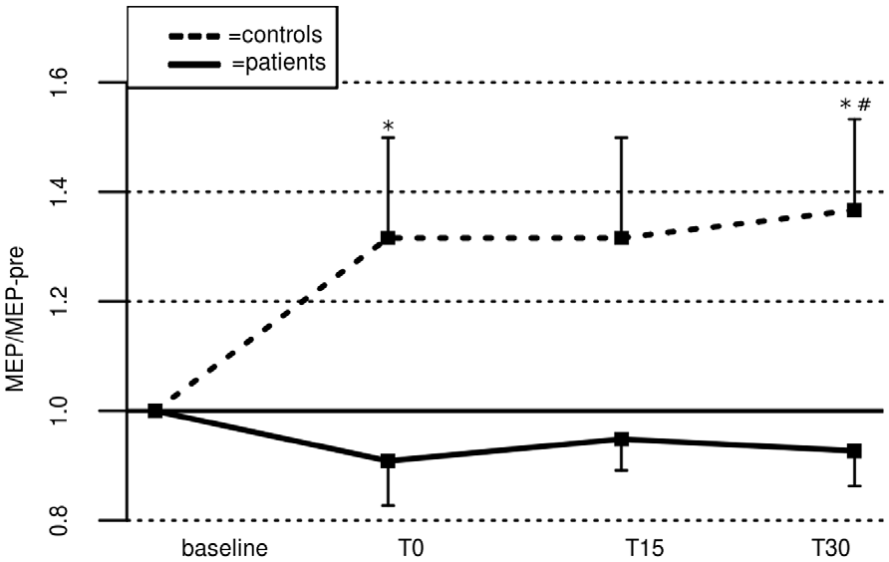
PAS-induced plasticity in patients and controls. Error bars indicate standard error of the mean. * indicate significant differences between groups (p<0.05; uncorrected), # indicates significant differences from baseline within groups (p<0.05, uncorrected).

Resting motor threshold (rMT) at baseline was 44±7% of maximum stimulator output in patients, and 45±5% in controls (difference n.s.). Five out of 16 patients, and 10 out of 16 controls responded to PAS with an MEP-increase (as indicated by mean MEP-changes following PAS, MEAN-LTP, **Figure 1**). MEAN-LTP was significantly lower in patients compared to controls (0.92±0.06 in patients vs 1.33±0.14 in controls; t_(30)_ = −2.76, p = 0.01, primary outcome). After PAS, no significant change of MEP amplitudes could be observed at any time point in the patient group. In controls, a significant increase of MEP size could be observed 30 minutes after PAS (T30) (p = 0.04; uncorrected for multiple comparisons, Figure 2). ANOVA_RM_ did not reveal a significant GROUP x TIME-Interaction, nor a significant main effect of TIME, but a main effect of GROUP (F_(1,29)_ = 6.41, p = 0.02). Post-hoc t-tests revealed significant differences between both groups at time point T0 (t_(30)_ = −2.25, p = 0.03) and T30 (t_(30)_ = −2.47, p = 0.02; uncorrected for multiple comparisons).

#### Differences between patients with left- vs. right-sided stenosis

Subgroup analysis of patients with left vs. right ICA-stenosis (n = 9 vs. n = 7) revealed no significant differences except for gender, with regard to demographical data, neuropsychological tests and TMS-measurements (**Table 3**).

**Table 3:** Left-sided vs. right-sided ICA-stenosis.

	ICA-Stenosis left (n = 9)	ICA-Stenosis right (n = 7)	p
**Age**	61±8	62±7	0.83
**Gender**	7 F, 2 M	1 F, 6 M	0.04
**Years of Education**	11.1±1.9	12.5±3	0.28
**MMSE**	28±1.4	27±1.5	0.83
**BDI**	8±4	6±3	0.23
**Verbal learning**	47±8	40±14	0.25
**Verbal memory**	9.3±2.9	8.4±3	0.55
**Working memory**	14±3	13±3	0.51
**Verbal fluency**	18±9	17±11	0.83
**rMT**	47±4	41±8	0.08
**MEAN-LTP**	0.91±0.05	0.93±0.14	0.95

MMSE: Mini mental state examination, BDI: Beck’s depression inventory, SD: standard deviation, rMT = resting motor threshold; groups were compared using two-sample t-tests, or Qui-Square tests (gender).

### Influence of Surgery on PAS/cognition

**Figure 3:**
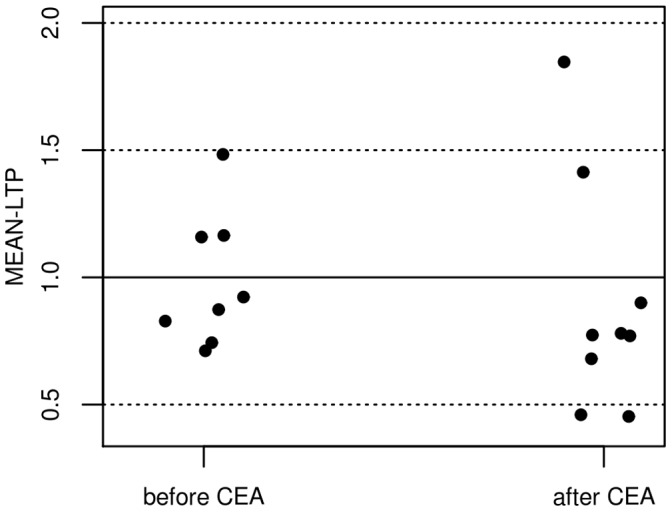
Influence of CEA on PAS-induced plasticity. Influence of CEA on PAS-induced plasticity is indicated by the mean MEP-changes (MEAN-LTP). Three out of 9 patients responded to PAS with the expected MEP-increase before CEA, 2 patients responded to PAS 6 months after CEA (difference n. s.).

All patients were asked to be re-assessed 6 months after CEA. Seven patients were lost to follow-up, due to one death (sepsis following pneumonia), and six patients that declined further examination due to time constraints. In sum, 9 out of 16 Patients (61±6 years, 3 women, 3 left-sided stenoses) agreed to participate in a follow-up assessment six months after successful surgery. CEA had no influence on any of the cognitive test scores (**Table 4**). Furthermore, CEA had no influence on rMT (before CEA: 43±8%, after CEA 43±6%, t_(8)_ = −0.09, p = 0.93, paired t-test) or on MEAN-LTP (before CEA: 0.93±0.10, after CEA: 0.90±0.15, t_(8)_ = 0.20; p = 0.85, paired t-test, **Figure 3**).

**Table 4:** Comparison of baseline and cognitive test-scores in patients before and 6 months after CEA.

Test	Patients before CEA (mean ± SD)	Patients after CEA (mean ± SD)	p
**MMSE**	27.9±1.1	28.3±1.8	0.47
**BDI**	6.6±4.1	6.8±5.9	0.89
**Verbal learning (AVLT (sum 1–5))**	46.3±8.1	46.4±11.1	0.96
**Verbal memory (AVLT delayed recall)**	7.9±2.8	9.6±2.8	0.12
**Working memory (WMS digit span, sum forward + backward**	13.7±2.7	12.6±2.3	0.12
**Verbal fluency**	17.4±8.9	17.0±5.9	0.92

MMSE: Mini mental state examination; BDI: Becks depression inventory; AVLT: Auditory verbal learning test; WMS: Wechsler memory scale; groups were compared using paired t-tests.

## Discussion

Two main findings emerged from the present study. First, we showed that rapid-onset cortical plasticity as well as verbal learning was reduced in patients with severe stenosis of the internal carotid artery as compared to age-matched controls. Second, no improvement of reduced rapid-onset cortical plasticity nor verbal learning was noted after surgical revascularisation.

### Cortical Plasticity is Impaired in Patients with Severe Stenosis of the Internal Carotid Artery

In patients with severe stenosis of the ICA the majority of studies reported reduced cognitive functions, specifically memory functions [26], [27], while executive functions were largely preserved [28].

Different mechanisms may underlie cognitive impairment in this condition. First, silent infarctions are known to increase the risk of developing cognitive decline [29], and occur frequently in patients with clinically asymptomatic ICA-stenosis [30]. Second, cognitive decline may be due to morphological and functional brain changes in chronic hypoperfusion conditions: Enzinger and colleagues [31] reported a decrease of hemispheric volume in patients with severe ICA-stenosis, as a possible morphological correlate of impaired cognition. More generally, brain hypoperfusion has been proposed to contribute to the onset of dementia, a hypothesis supported by the fact that patients with heart failure and general hypoperfusion experience accelerated cognitive decline [32]. Based on animal studies, it has been hypothesised that impaired cognition may be caused by reduced brain plasticity following hypoperfusion. In rats, reduced LTP in the hippocampus as well as attenuated learning and memory could be demonstrated in chronic hypoperfusion [33]. These changes occurred even in the absence of obvious histological changes [34]. Also in humans, it has been shown that learning and memory formation are related to LTP-like mechanisms [35]. Our study extends this finding by demonstrating that LTP-like rapid-onset cortical plasticity, as assessed within the motor cortex, is impaired in the hemisphere of humans affected by chronic hypoperfusion. These findings now need to be corroborated in a larger cohort. Corresponding to the changes in rapid-onset cortical plasticity, the patients exhibited decreased learning abilities on cognitive testing, while working memory and verbal fluency did not show this decline, probably due to the fact that these functions are less dependent on LTP-like mechanisms [36], [37].

### Reperfusion Dos not Improve Cortical Plasticity

Some studies reported improvement of cognitive functions after CEA [38], while others found evidence for cognitive decline [5]. Better cognitive performance may be related to increased perfusion of the affected hemisphere [39]. In contrast, microinfarctions frequently occur during revascularisation, and may worsen cognitive functions [40]. It should be noted, however, that a recent study found no association between cognitive decline and microinfarctions during CEA [41]. Furthermore, decreased cortical plasticity due to silent strokes in our patients is unlikely, since manifest stroke has been shown to enhance rapid-onset cortical plasticity [12], [42].

Impaired cognition after CEA may also be due to hypoperfusion during cross-clamp time. In fact, larger decline has been noted in patients with higher cross-clamp time and thus longer hypoperfusion during surgery [43]. All patients included into the present study received an intraluminar shunt during clamping to minimize hypoperfusion time. Thus, a worsening of cognitive function due to hypoperfusion seems unlikely in our cohort.

No improvement of cognition and rapid-onset cortical plasticity six months after CEA was observed in our cohort either. Thus, CEA may not be an be an appropriate tool to restore cortical plasticity and subsequently cognitive functions, and other strategies to improve cognitive function should be evaluated in these patients. Interestingly, in animal studies, flavonoids led to a restoration of LTP in chronic hypoperfusion [44]. In humans, there are first indications that flavonoids may have beneficial functions on cognition in general [45], and their impact on both rapid-onset cortical plasticity and cognition in patients with severe ICA stenosis should be tested.

### Factors that may Influence Cortical Plasticity

Rapid-onset cortical plasticity displays large inter- and intra-subject variability [46], due to age [25], [46], gender [47], genetic factors [17], attention [48], time of day [49], and level of previous physical activity [13], among others.

To reduce variability, gender [47] and attention [48] were held constant between both groups. Thus, differences between both groups according to rapid-onset cortical plasticity are unlikely due to the time of day the measurements were performed. Moreover, both groups were matched with regard to age, since previous studies that included middle-aged or elderly participants showed reductions in PAS-induced plasticity [25], [46], compared to results known for young subjects. Our findings of small but significant PAS-effect in the elderly controls 30 min after PAS only was in line with these previous studies. Heterogeneous results between studies, particularly with regard to time-line of MEP changes after PAS induction, might stem from slight variations in PAS protocols. In the present study, controls only displayed significant increase of MEP 30 minutes after PAS, similar to Müller et al [50], Weise et al [23], and List et al [51] that also found most prominent changes 30 min after PAS, but different from Conde et al [52] that found most pronounced changes directly after PAS. Given the variations in PAS-protocols, direct comparison between individual studies remains difficult.

So far, little is known with regard to the impact of cardiovascular risk factors on rapid-onset cortical plasticity, but the available evidence does not point to a general reduction: Hypertension-induced cerebral microangiopathy does not lead to decreased rapid-onset cortical plasticity [51]. Nicotine consumption even led to slightly enhanced cortical plasticity [53]. Therefore the presence of cardiovascular risk factors is rather unlikely to explain the differences between both groups.

Note also that group sizes in the present study were similar to or even greater than in previous studies that found significant differences between specific patient groups and healthy controls [23], [13], [12]. Consequently, we were able to ascertain a significant difference for rapid-onset cortical plasticity between groups.

### Limitations

One limitation of our study is the rather small sample size. This might explain the lack of significant association between TMS-measurements and cognitive test results. Second, the impact of the ICA-stenosis as such, as well as the subsequent effect of the CEA procedure, on the metabolism of the ipsilateral hemisphere, e.g. by using nuclear imaging techniques [54], was not assessed. Furthermore, a more extensive battery of neurophysiological assessments for cortical plasticity, e.g., using transcranial direct current stimulation [55], should be employed to corroborate the effects of chronic hypoperfusion and CEA on cortical plasticity. Third, only the hemisphere ipsilateral to the ICA stenosis was examined. In future studies, ipsilateral and contralateral hemispheres before and after intervention should be assessed for rapid- onset cortical plasticity in patients, and a re-assessment after 6 months of one hemisphere should be conducted in healthy participants to demonstrate test-re-test reliability of the protocol in general.

### Conclusion

Our study demonstrates that chronic hypoperfusion reduces rapid-onset cortical plasticity, which may contribute to the decline in cognition found in patients with severe ICA stenosis, a finding to be substantiated in a larger group of patients. Since surgical revascularisation, although reducing stroke risk, did not restore rapid-onset cortical plasticity, additional therapeutic strategies are needed. Pharmacological and dietary agents [45], [56] or endogenous up-regulation of brain plasticity via physical activity [13] are promising means for cognitive rehabilitation that need to be tested in future studies.

## References

[1] Petty GW, Brown RD, Whisnant JP, Sicks JD, O’Fallon WM (1999). Ischemic stroke subtypes: a population-based study of incidence and risk factors.. *Stroke*.

[2] Goldstein LB, Adams R, Alberts MJ, Appel LJ, Brass LM (2006). Primary prevention of ischemic stroke: a guideline from the American Heart Association/American Stroke Association Stroke Council: cosponsored by the Atherosclerotic Peripheral Vascular Disease Interdisciplinary Working Group; Cardiovascular Nursing Council; Clinical Cardiology Council; Nutrition, Physical Activity, and Metabolism Council; and the Quality of Care and Outcomes Research Interdisciplinary Working Group: the American Academy of Neurology affirms the value of this guideline.. *Stroke*.

[3] Béjot Y, Aboa-Eboulé C, Durier J, Rouaud O, Jacquin A (2011). Prevalence of early dementia after first-ever stroke: a 24-year population-based study.. *Stroke*.

[4] Silvestrini M, Paolino I, Vernieri F, Pedone C, Baruffaldi R (2009). Cerebral hemodynamics and cognitive performance in patients with asymptomatic carotid stenosis.. *Neurology*.

[5] Aharon-Peretz J, Tomer R, Gabrieli I, Aharonov D, Nitecki S (2003). Cognitive performance following endarterectomy in asymptomatic severe carotid stenosis.. *Eur J Neurol*.

[6] Heyer EJ, Adams DC, Solomon RA, Todd GJ, Quest DO (1998). Neuropsychometric changes in patients after carotid endarterectomy.. *Stroke*.

[7] Witt K, Börsch K, Daniels C, Walluscheck K, Alfke K (2007). Neuropsychological consequences of endarterectomy and endovascular angioplasty with stent placement for treatment of symptomatic carotid stenosis: a prospective randomised study.. *J Neurol*.

[8] Bonati LH, Jongen LM, Haller S, Flach HZ, Dobson J (2010). New ischaemic brain lesions on MRI after stenting or endarterectomy for symptomatic carotid stenosis: a substudy of the International Carotid Stenting Study (ICSS).. *Lancet Neurol*.

[9] Stefan K, Kunesch E, Cohen LG, Benecke R, Classen J (2000). Induction of plasticity in the human motor cortex by paired associative stimulation.. *Brain*.

[10] Wolters A, Sandbrink F, Schlottmann A, Kunesch E, Stefan K (2003). A temporally asymmetric Hebbian rule governing plasticity in the human motor cortex.. *J Neurophysiol*.

[11] Rioult-Pedotti MS, Friedmann D, Donoghue JP (2000). Learning-induced LTP in neocortex.. *Science*.

[12] List J, Duning T, Meinzer M, Kürten J, Schirmacher A (2011). Enhanced rapid-onset cortical plasticity in CADASIL as a possible mechanism of preserved cognition.. *Cereb Cortex*.

[13] Cirillo J, Lavender AP, Ridding MC, Semmler JG (2009). Motor cortex plasticity induced by paired associative stimulation is enhanced in physically active individuals.. *J Physiol*.

[14] Battaglia F, Wang HY, Ghilardi MF, Gashi E, Quartarone A (2007). Cortical plasticity in Alzheimer’s disease in humans and rodents.. *Biol Psychiatry*.

[15] Battaglia F, Quartarone A, Rizzo V, Ghilardi MF, Di Rocco A (2008). Early impairment of synaptic plasticity in patients with Down’s syndrome.. *Neurobiol Aging*.

[16] Frantseva MV, Fitzgerald PB, Moller B, Daigle M, Daskalakis ZJ (2008). Evidence of impaired long-term potentiation in schizophrenia and ist relationship to motor skill learning.. *Cereb Cortex*.

[17] Witte AV, Kurten J, Jansen S, Schirmacher A, Brand E (2012). Interaction of BDNF and COMT Polymorphisms on Paired-Associative Stimulation-Induced Cortical Plasticity.. *J Neurosci*.

[18] MRC european carotid surgery trial (1991). Interim results for symptomatic patients with severe (70–99%) or with mild (0–29%) carotid stenosis. European carotid surgery trialists’ collaborative group.. *Lancet*.

[19] Folstein MF, Folstein SE, McHugh PR (1975). ”Mini-mental state”. A practical method for grading the cognitive state of patients for the clinician.. J Psychiatr Res.

[20] Hautzinger M, Bailer M, Worall H, Keller F (1994). Beck-Depressions-Inventar (BDI).. Bern: Huber.

[21] Oldfield RC (1971). The assessment and analysis of handedness: the Edinburgh inventory.. *Neuropsychologia*.

[22] Lezak MD (2004). Neuropsychological assessment.. New York, Oxford: Oxford University Press.

[23] Weise D, Schramm A, Stefan K, Wolters A, Reiners K (2006). The two sides of associative plasticity in writer’s cramp.. *Brain*.

[24] R Development Core-Team (2010). R: A Language and Environment for Statistical Computing.. Vienna (Austria): R Foundation for Statistical Computing.

[25] Müller-Dahlhaus JF, Orekhov Y, Liu Y, Ziemann U (2008). Interindividual variability and age-dependency of motor cortical plasticity induced by paired associative stimulation.. *Exp Brain Res*.

[26] Romero JR, Beiser A, Seshadri S, Benjamin EJ, Polak JF (2009). Carotid Artery Atherosclerosis, MRI Indices of Brain Ischemia and Cognitive Impairment.. *Stroke* 40; 1590–1596.

[27] Mathiesen EB, Waterloo K, Joakimsen O, Bakke SJ, Jacobsen EA (2004). Reduced neuropsychological test performance in asymptomatic carotid stenosis: The Tromsø Study.. *Neurology*.

[28] Landgraff NC, Whitney SL, Rubinstein EN, Yonas H (2010). Cognitive and physical performance in patients with asymptomatic carotid artery disease.. *J Neurol*.

[29] Arvanitakis Z, Leurgans SE, Barnes LL, Bennett DA, Schneider JA (2011). Microinfarct pathology, dementia, and cognitive systems.. *Stroke*.

[30] Norris JW, Zhu CZ (1992). Silent stroke and carotid stenosis.. *Stroke*.

[31] Enzinger C, Ropele S, Gattringer T, Langkammer C, Schmidt R (2010). High-grade internal carotid artery stenosis and chronic brain damage: a volumetric magnetic resonance imaging study.. *Cerebrovasc Dis*.

[32] Zuccalà G, Onder G, Pedone C, Carosella L, Pahor M (2001). Hypotension and cognitive impairment: Selective association in patients with heart failure.. *Neurology*.

[33] Li S, He Z, Guo L, Huang L, Wang J (2010). Behavioral alterations associated with a down regulation of HCN1 mRNA in hippocampal cornus ammon 1 region and neocortex after chronic incomplete global cerebral ischemia in rats.. *Neuroscienc*e.

[34] Mori K, Yoshioka M, Suda N, Togashi H, Matsumoto M (1998). An incomplete cerebral ischemia produced a delayed dysfunction in the rat hippocampal system.. *Brain Res*.

[35] Ziemann U, Ilić TV, Iliać TV, Pauli C, Meintzschel F (2004). Learning modifies subsequent induction of long-term potentiation-like and long-term depression-like plasticity in human motor cortex.. *J Neurosci*.

[36] Khan ZU, Muly EC (2011). Molecular mechanisms of working memory.. *Behav Brain Res* [epub ahead of print].

[37] Eisenberg DP, Berman KF (2010). Executive function, neural circuitry, and genetic mechanisms in schizophrenia.. *Neuropsychopharmacology*.

[38] Fearn SJ, Hutchinson S, Riding G, Hill-Wilson G, Wesnes K (2003). Carotid endarterectomy improves cognitive function in patients with exhausted cerebrovascular reserve.. *Eur J Vasc Endovasc Surg*.

[39] Incalzi RA, Gemma A, Landi F, Pagano F, Capparella O (1997). Neuropsychologic effects of carotid endarterectomy.. *J Clin Exp Neuropsychol*.

[40] Ghogawala Z, Westerveld M, Amin-Hanjani S (2008). Cognitive outcomes after carotid revascularization: the role of cerebral emboli and hypoperfusion.. *Neurosurgery*.

[41] Wasser K, Pilgram-Pastor SM, Schnaudigel S, Stojanovic T, Schmidt H (2011). New brain lesions after carotid revascularization are not associated with cognitive performance.. *J Vasc Surg*.

[42] Castel-Lacanal E, Marque P, Tardy J, de Boissezon X, Guiraud V (2009). Induction of cortical plastic changes in wrist muscles by paired associative stimulation in the recovery phase of stroke patients.. *Neurorehabil Neural Repair*.

[43] Heyer EJ, DeLaPaz R, Halazun HJ, Rampersad A, Sciacca R (2006). Neuropsychological dysfunction in the absence of structural evidence for cerebfral ischemia after uncomplicated carotid endarterectomy.. *Neurosurgery*.

[44] Xu B, Li XX, He GR, Hu JJ, Mu X (2010). Luteolin promotes long-term potentiation and improves cognitive functions in chronic cerebral hypoperfused rats.. *Eur J Pharmacol*.

[45] Nurk E, Refsum H, Drevon CA, Tell GS, Nygaard HA (2009). Intake of flavonoid-rich wine, tea, and chocolate by elderly men and women is associated with better cognitive test performance.. *J Nutr*.

[46] Kang JS, Terranova C, Hilker R, Quartarone A, Ziemann U (2010). Deficient homeostatic regulation of practice-dependent plasticity in writer’s cramp.. *Cereb Cortex*.

[47] Tecchio F, Zappasodi F, Pasqualetti P, De Gennaro L, Pellicciari MC (2008). Age dependence of primary motor cortex plasticity induced by paired associative stimulation.. *Clin Neurophysiol*.

[48] Stefan K, Wycislo M, Classen J (2004). Modulation of associative human motor cortical plasticity by attention.. *J Neurophysiol*.

[49] Sale MV, Ridding MC, Nordstrom MA (2008). Cortisol inhibits neuroplasticity induction in human motor cortex.. *J Neurosci*.

[50] Muller JF, Orekhov Y, Liu Y, Ziemann U (2007). Homeostatic plasticity in human motor cortex demonstrated by two consecutive sessions of paired associative stimulation.. *Eur J Neurosci*.

[51] List J, Duning T, Kurten J, Deppe M, Wilbers E (2012). Cortical plasticity is preserved in nondemented older individuals with severe ischemic small vessel disease.. *Hum Brain Mapp* [epub ahead of print].

[52] Conde V, Vollmann H, Sehm B, Taubert M, Villringer A (2012). Cortical thickness in primary sensorimotor cortex influences the effectiveness of paired associative stimulation.. *Neuroimage*.

[53] Thirugnanasambandam N, Grundey J, Adam K, Drees A, Skwirba AC (2011). Nicotinergic impact on focal and non-focal neuroplasticity induced by non-invasive brain stimulation in non-smoking humans.. *Neuropsychopharmacology*.

[54] Matsubara S, Moroi J, Suzuki A, Sasaki M, Nagata K (2009). Analysis of cerebral perfusion and metabolism assessed with positron emission tomography before and after carotid artery stenting. Clinical article.. *J Neurosurg*.

[55] Stagg CJ, Nitsche MA (2011). Physiological basis of transcranial direct current stimulation.. *Neuroscientist*.

[56] Spencer JPE, Vauzour D, Rendeiro C (2009). Flavonoids and cognition: the molecular mechanisms underlying their behavioural effects.. *Arch Biochem Biophys*.

